# Uncovering hidden depression: the critical role of depression screening in sleep disorders at U.S. sleep centers

**DOI:** 10.3389/fpsyt.2025.1449360

**Published:** 2025-04-25

**Authors:** Silvia Daccò, Massimiliano Grassi, Zach Wolpe, Melissa Bruner, Daniela Caldirola, Giampaolo Perna, Archie Defillo

**Affiliations:** ^1^ Department of Biomedical Sciences, Humanitas University, Milan, Italy; ^2^ Personalized Medicine Center for Anxiety and Panic Disorders, Humanitas San Pio X Hospital, Milan, Italy; ^3^ Clinical Research and Development Department, Medibio Limited, Savage, MN, United States

**Keywords:** depression, screening, sleep centers, sleep disorders, Patient Health Questionnaire 9, Mini-International Neuropsychiatric Interview

## Abstract

**Introduction:**

Depression and sleep disorders are strongly linked, with sleep centers (SCs) reporting depressive symptoms in up to 63% of patients and depression diagnoses in 22%. Despite this, routine depression screening is not standard in SCs. This study aimed to identify patients meeting criteria for a current major depressive episode (cMDE) among those self-reporting clinically significant depressive symptoms (CSDS) in U.S. SCs.

**Method:**

This retrospective sub-analysis of an ongoing multicenter trial included 147 adults (22–75 years) referred for sleep disorder evaluation. Participants underwent psychiatric assessments using the Mini-International Neuropsychiatric Interview and Patient Health Questionnaire-9 (PHQ-9), with CSDS defined as PHQ-9 ≥10. Descriptive statistics were compared between patients with/without a confirmed cMDE using non-parametric tests. Additional Mann-Whitney U tests assessed sleep characteristics by cMDE, major depressive disorder (MDD), and bipolar disorder (BD) (statistical significance, p < 0.05).

**Results:**

Of 147 patients, 57 (38.8%) had a PHQ-9 score ≥10. Among them, 23 (40.3%) were diagnosed with cMDE: 17 (29.8%) met cMDD criteria, and 6 (10.5%) had BD, type I. A significantly lower cMDE prevalence was observed in patients without CSDS. Sleep characteristics showed no significant differences except for a lower N3 percentage in cMDE. BD, type I was associated with higher obstructive sleep apnea comorbidity compared to cMDD.

**Discussion:**

Our findings suggest major depression prevalence in SCs is five times higher than in the general population, highlighting the need for routine depression screening and psychiatric confirmation. This also aids in identifying comorbidities and fostering tailored interventions to improve outcomes.

## 1 Introduction

Depression is a leading cause of disease burden, disability, persistent impairment in quality of life, and increased risk of suicide worldwide (1–4). In 2021, recent past-year prevalence estimates revealed that 21.0 million adults in the United States experienced at least one major depressive episode (MDE), accounting for 8.3% of the total adult population. (5). Other findings showed increases in past-year prevalence of depression in the U.S. population from 6.6% in 2005 to 9.2% in 2020 (6, 7).

Recognizing the psychosocial burden of depression, professional organizations, and international guidelines have included routine screening for depression in their preventive health recommendations starting from primary care to specialty medical settings, (8–11).

However, depression is likely to be missed by primary care providers in the U.S. In 2005, the national depression screening rate during adult visits to primary care physicians was just 1.4%; unfortunately, the increase over the years has been limited, achieving only approximately 3% in 2020 (11–16). Consistent with this, recent studies reported that over 50% of depressed patients in primary care are still unrecognized and undertreated (12, 17–19).

For these reasons, the scientific and clinical community, as well as the World Health Organization (WHO), have repeatedly highlighted that more attention should be devoted to detecting current depression not only in the general population but also in outpatients with different medical conditions, with the final aim of improving functional and clinical outcome (1, 20, 21).

Depression is strongly associated with sleep impairment (22) and represents a significant risk factor for developing sleep disorders. Studies indicate that approximately 40% of participants who reported severe depressive symptoms at baseline developed insomnia within a 12-month follow-up period (23). Conversely, sleep problems and complaints often appear before other depressive symptoms (24), and subjective sleep quality worsens even before the onset of a depressive episode in recurrent depression (25). Several longitudinal studies and meta-analyses have shown that non-depressed individuals with insomnia have a twofold increased risk of developing subsequent depression within at least 12 months compared to those without sleep difficulties (26–29). In addition, the comorbidity of depression and sleep disturbance or disorders have detrimental effects on the depression clinical manifestation and outcome, resulting in increased severity of clinical depression symptoms (30), worse treatment response (31), higher risk of relapse (31), and increased likelihood —ranging from 1.24 to 2.41 times—of suicidal ideation and suicide attempts compared to those without sleep disorders (32–34). In addition, meta-analyses indicate that insomnia is an independent risk factor for suicide and this association persists even after accounting for the presence of depressive disorder and controlling for symptom intensity (35).

Despite the well-documented clinical significance of the sleep-depression association, the available literature suggests that community sleep centers (SCs) in the U.S. do not routinely include depression screenings as part of their standard care practices.

In SCs, the few available data reported up to 63% prevalence of depressive symptoms using self-rating questionnaires and approximately up to 22% prevalence of a psychiatric interview-based depression diagnosis (36, 37).

Studies reported a range of prevalence rates for depressive symptoms in patients with obstructive sleep apnea (OSA), typically falling between 32% and 50% (38–40). Additionally, major depression has been diagnosed in 17% to 40% of individuals with OSA (41–44). Similarly, a meta-analysis revealed that the overall pooled prevalence of depression or depressive symptoms in patients with narcolepsy was 32% (45). Approximately three-quarters of individuals with delayed sleep phase syndrome had a past or current history of depression and 14% reported moderate-severe depressive symptoms (46, 47). In clinical samples, from 18 to 71% of patients with restless legs syndrome or periodic limb movements have been diagnosed with depression (48), while in population-based samples, 12-month major depression prevalence has been reported in 9.5% of participants (49).

Given the high comorbidity between sleep disorders and depression, this study explored the importance of depression screening in the U.S. SCs. Our research aimed to identify patients who met the criteria for current MDE (cMDE) among those who presented self-reported clinically significant depressive symptoms in several U.S. SCs.

## 2 Material and methods

### 2.1 Study design

This study is a retrospective sub-analysis of a larger, ongoing, single-arm, prospective, multicenter trial titled “Investigation of the Likelihood of a Current Major Depressive Episode (cMDE) in Individuals Referred to Sleep Clinics for Polysomnography (PSG) Assessment Using the MEB-001 Device.” The primary aim of the mentioned trial is to develop a machine-learning algorithm capable of screening individuals who may have cMDE from PSG data, including electroencephalographic (EEG) signals and heart rate variability (HRV).

This ongoing study was approved by Western Institutional Review Board (Puyallup, WA, USA) (Approval No. 1230523) and conducted in accordance with the principles of the United States Food and Drug Administration Code of Federal Regulations Part 2, Good Clinical Practice (50), and the Declaration of Helsinki (51). The study protocol has been registered in ClincialTrials.gov register (registration number NCT0570822).

### 2.2 Subjects

This sub-analysis adheres to the inclusion and exclusion criteria of the larger, ongoing algorithm development study. It includes 147 consecutively recruited adult patients aged between 22 and 75 years (55 females, 37.4%) referred to the SC for a sleep study to undergo a PSG for sleep-awake disorder investigation. Individuals under 22 were excluded to avoid potential electrophysiological instability associated with the transition from late adolescence to early adulthood. Participants over 75 were excluded to minimize the impact of age-related changes mainly in HRV on the algorithm’s performance. All included patients underwent a full-night diagnostic PSG study as prescribed. They were eligible if they could read and understand the study instructions, adhere to the procedures, and provide informed consent. Exclusion criteria included having a pacemaker, a history of a heart transplant, or having previously undergone a full-night CPAP titration study.

Patients were recruited from 13 community SCs, based in different geographic areas in the U.S. (i.e., Minnesota, Texas, North Caroline, South Caroline, Ohio, Florida), to guarantee the geographic diversity necessary to obtain a representative sample.

### 2.3 Study procedures

According to the SC procedures, on the night of the PSG, the sleep clinician or designee invited the potential subject to participate in this study and provided him/her with an informed consent form. After having signed the informed consent, the subject was assigned an identity code (ID) and moved to the PSG room where a psychiatric assessment was administered before undergoing the PSG as prescribed, according to good clinical practice. The psychiatric assessment was composed of a clinician-administered interview for the psychiatric cMDE diagnosis and a self-administered evaluation for the determination of depressive symptom severity. To reduce potential discrepancies between clinician-administered and self-reported assessments, we selected the Mini-International Neuropsychiatric Interview (MINI), Adult Version (52) and the Patient Health Questionnaire-9 (PHQ-9) (53–55), respectively. Both instruments assess the criteria for cMDE according to the Diagnostic and Statistical Manual of Mental Disorders, Fifth Edition (DSM-5), focusing on the core depressive symptoms. This choice enhances the reliability of the comparison, reducing the risk of inconsistent depression assessments and minimizing the possibility of differing clinical characterizations.

#### 2.3.1 The MINI interview assessment

Ten licensed nurse practitioners with experience in psychiatric conditions participated in the study across various geographical regions. Each conducted the clinician-administered MINI interview at the center to which they were assigned. Each nurse first verified the subject’s demographic information, including age, gender, race and ethnicity, marital status, education, and body mass index, to update the medical records. Additionally, the nurse collected details about the subject’s lifestyle.

The MINI is a brief structured diagnostic interview for the major psychiatric disorders in the DSM-5 (56) and International Classification of Diseases, Tenth Edition (ICD-10) (57), widely used in mental health settings worldwide. Validation and reliability studies of the MINI showed a sensitivity of 0.96, specificity of 0.88, positive predictive value of 0.87, negative predictive value of 0.97, inter-rater Kappa 1.00, and test/retest Kappa 0.87 in major depressive disorder (MDD) diagnosis (52) when compared to the clinician-administered semi-structured interviews (i.e., Structured Clinical Interview (SCID) (58)). In bipolar diagnosis (BD) diagnosis, the MINI showed a sensitivity of 0.82, specificity of 0.95, positive predictive value of 0.63, negative predictive value of 0.98, and an inter-rater Kappa of 0.67 in detecting a current manic episode, while a sensitivity of 0.81, specificity of 0.94, positive predictive value of 0.76, negative predictive value of 0.95, and an inter-rater Kappa 0.73, in detecting a past manic episode when compared to the clinician-administered SCID (52). The MINI reports also a good agreement even when administered by non-mental health experts (52, 59, 60). The MINI has been used as a reference for the PHQ-9 validation. Compared to the MINI, the PHQ-9 at the cut-off score of 10 showed a sensitivity of 0.74 (0.67 to 0.79) and specificity. 0.89 (0.86 to 0.91) (61).

The MINI assessment was conducted using a tablet-based web interface, facilitating data capture through the Proem platform, an online tool for mental health screening and diagnosis (62). The Proem system automatically processed MINI responses and generated diagnoses based on MINI scoring rules (63).

The involved nurses were trained according to the MINI training procedures required by the MINI’s author and copyright holder (Harm Research Institute, no date) (64). They attended an approximately 1-hour training session on MINI instructions and administration by two licensed MINI trainers (MB and TY). Since the administration was conducted via the Proem platform, the training included a specific focus on the platform’s structure and functionality. An additional 1-hour training session was held on a separate day, focusing on the DSM-5 criteria for cMDE. This session included detailed explanations and clinical examples for each DSM-5 criterion, along with their corresponding MINI questions. Each nurse attended a 30-minute role-playing session to practice the MINI administration and make sure they didn’t have any issues or questions with the assessment and the technology. To increase the reliability of the nurses’ assessment, during the 30-minute role-playing, the trainers simulated two clinical patients suffering from cMDE with and without past manic symptoms, respectively. The difference between manic and hypomanic symptoms was also highlighted and discussed after the role-playing. The role-playing sessions were unstructured, with various cases presented to different nurses. In addition, at the end of the 30-minute role-playing session, the suicidality protocol for patients identified as being at risk of suicide was presented. The role-playing practice was performed on the same day as one of the two training sessions or on a different day, according to the availability of the nurse. Following the MINI administration and DSM-5 criteria training sessions, the participating nurses completed a 1-hour ICH Good Clinical Practice (GCP) E6 (R2) course to obtain the corresponding certification.

Due to the wide geographic distribution of the SCs involved in the study, all the training sessions were conducted online.

#### 2.3.2 The PHQ-9

The self-administered assessment PHQ-9 was administered in the digital format (65). To complete this assessment, the subjects logged onto the Proem platform using a tablet and completed the PHQ-9. An automated score was generated by Proem platform, using the PHQ-9 scoring rules (54).

The PHQ-9 (53–55) is a validated 9-item self-report questionnaire widely used in depression assessment (66). As the PHQ-9 has demonstrated clinical utility and accuracy, it has been recommended by the United States Preventive Services Task Force and others for depression severity assessment and screening in primary care and other medical settings (9, 66–69). In addition, the PHQ-9 has been considered acceptable and reliable to use in the major sociodemographic groups in the U.S., without significant differences in performance across groups (70, 71).

Its 9 items align with the 9 DSM-5 criteria for a cMDE (72). A recent and comprehensive Individual Participant Data Meta-analysis identified a cutoff of 10 or greater as a threshold that “draws attention to a possible clinically significant condition”, namely a possible cMDE, with a sensitivity and specificity ranging from 0.67 to 0.88 and from 0.86 to 0.88, respectively compared to reference standards (i.e., semi or fully structured psychiatric diagnostic interviews).

Consistently, a score of ≥ 10 has been associated with an increased risk of major depression more than 2.6 times (54). Overall, based on the large body of scientific evidence concerning PHQ-9, this cutoff threshold (≥ 10) approach is advised as the most reliable for screening use in clinical practice and clinical trials (73).

#### 2.3.3 PSG

The PSG study was performed after the clinician-administered and self-administered assessments according to good clinical practice and following the American Academy of Sleep Medicine (AASM) guidelines (74) for the administration and the scoring procedures. Different PSG devices have been used according to the system availability of the involved SC. Sleep disorders or sleep-related symptoms were identified the following days according to the SC’s procedures according to the AASM International Classification of Sleep Disorders (AASM-ICSD) and included in the medical record.

### 2.4 Statistical analysis

To assess the normality of the data, we conducted the Shapiro-Wilk test. Since some continuous variables within certain groups exhibited significant deviations from normality (p < 0.05), non-parametric statistical tests were applied for further analyses.

Descriptive analyses reported the median and interquartile range for continuous variables, while categorical variables were summarized using frequencies and percentages. Prevalence rates of sleep disorders/sleep-related symptoms, cMDE, current major depressive disorder (cMDD), and BD were reported as percentages. To examine differences in socio-demographic and clinical variables between patients with PHQ-9 ≥ 10 with and without a confirmed cMDE, we performed the Mann-Whitney U test for continuous variables, Fisher’s Exact Test for dichotomous variables, and Chi-squared tests for nominal variables.

Given the well-established bidirectional relationship between depression and sleep disturbances, we extended our analysis to include a comparative perspective for completeness. Specifically, we examined whether clinically significant depressive symptoms (PHQ-9 ≥ 10), with or without a cMDE, as well as the two diagnoses—cMDD and BD—differ in terms of sleep characteristics. We used the Mann-Whitney U analysis to compare PSG indices between subjects in the following groups: (1) PHQ-9 ≥ 10 without a cMDE diagnosis vs. PHQ-9 ≥ 10 with cMDE and (2) PHQ-9 ≥ 10 with cMDD vs. PHQ-9 ≥ 10 with cMDE in BD. The PSG indices analyzed included total sleep time, sleep efficiency index, apnea-hypopnea index (AHI) events per hour, percentage of time with peripheral oxygen saturation <88%, respiratory arousal index, periodic limb movements arousal index, spontaneous arousal index, total arousal index, percentage of time spent awake, and time spent in Non-Rapid Eye Movement stage 1, 2, 3 (N1, N2, N3), and Rapid Eye Movement (REM), REM latency, wake after sleep onset, and sleep onset latency. The percentage of time spent in each sleep stage was calculated from the moment the subject fell asleep. Statistical significance was set at p < 0.05.

We also reported the distribution of sleep disorders and sleep-related symptoms among patients with clinically significant depressive symptoms (PHQ-9 ≥ 10) who had a confirmed diagnosis of cMDD or BD. Due to the small sample size, statistical significance was not calculated for these distributions.

## 3 Results

Descriptive analysis of sociodemographic and clinical variables is reported in **Table 1**. The comparison between patients with PHQ-9 scores ≥ 10, with or without a cMDE, revealed no statistically significant differences, except for a higher number of Asian patients with a confirmed cMDE (Fisher’s Exact Test, p = 0.025).

**Table 1:** Sociodemographic and clinical characteristics of the whole sample (n=147) and a comparison between patients with and without a confirmed cMDE.

		Patients with PHQ-9 ≥ 10 without cMDE (n=34)	Patients with PHQ-9 ≥ 10 with cMDE (n=23)	Mann-Whitney U test^a^/Fisher’s Exact Test^b^/Chi-squared test^c^, p- value
Sociodemographic variables				
*Age, years; median (25^th^-75^th^ percentile)*	38 (30-51)	33 (27-39)	34 (27-47)	351.000, 0.641^a^
*Gender, females; n (%)*	55 (37.414)	22 (66.666)	12 (52.173)	0.254^b^
***Race, n(%)***				
*Hispanic*	16 (10.884)	4 (12.121)	4 (17.391)	0.715^b^
*White*	105 (71.428)	25 (75.757)	14 (60.869)	0.230^b^
*Black/African*	30 (20.408)	6 (18.181)	7 (30.434)	0.350^b^
*Asian*	6 (4.081)	0 (0)	4 (17.391)	0.025^b^*
*Native American*	5 (3.401)	0 (0)	1 (4.347)	0.418^b^
*Native Hawaiian*	4 (2.721)	1 (3.030)	2 (8.695)	0.565^b^
*Other*	8 (5.442)	4 (12.121)	1 (4.347)	0.386^b^
***Marital status, n(%)***				
*Married*	99 (67.347)	16 (48.484)	15 (65.217)	0.418^c^
*Never Married*	30 (20.408)	13 (39.393)	6 (26.086)	
*Separated*	1 (0.680)	1 (3.030)	0 (0)	
*Widowed*	3 (2.041)	0 (0)	0 (0)	
*Divorced*	8 (5.442)	1 (3.030)	1 (4.347)	
*Living with partner*	1 (0.680)	0 (0)	1 (4.347)	
*Marital Status: Prefer not to answer*	5 (3.401)	2 (6.060)	0 (0)	
***Education, n(%)***				
*High School Diploma*	31 (21.088)	7 (21.212)	7 (30.434)	0.391^c^
*Associate Degree*	21 (14.286)	3 (9.090)	5 (21.739)	
*Some Undergraduate*	18 (12.245)	3 (9.090)	4 (17.391)	
*Bachelor’s Degree*	35 (23.810)	10 (30.303)	5 (21.739)	
*Some Graduate*	11 (7.482)	3 (9.090)	1 (4.347)	
*Graduate Degree*	27 (18.367)	6 (18.181)	1 (4.347)	
*Other*	4 (2.721)	1 (3.030)	0 (0)	
***Employment status, yes; n (%)***				
*Employed*	112 (76.190)	25 (75.757)	17 (73.913)	0.550^c^
*Retired*	16 (10.884)	3 (9.090)	1 (4.347)	
*Unemployed*	18 (12.245)	4 (12.121)	5 (21.739)	
*Missing value*	1 (0.680)	1 (3.030)	0 (0)	
Clinical variables				
*PHQ9*≥*10; n (%)*	57 (38.775)	34 (59.649)	23 (40.350)	<0.0001^b^*
*PHQ9<10; n (%)*	90 (61.224)	85 (94.444)	5 (5.555)	
*BMI; median (25^th^-75^th^ percentile)*	30.105 (26.437-36.214)	33.670 (26.583-39.413)	33.670 (26.431-35.150)	458.000, 0.194^a^
**AASM-ICSD based sleep disorders/sleep-related symptoms performed through the PSG, n (%)**				
*OSA*	103 (70.068)	21 (61.764)	13 (56.521)	0.785^b^
*Sleep-Related Hypoxemia Disorder*	25 (17.006)	5 (14. 705)	4 (17.391)	1^b^
*PLM disorder*	23 (15.646)	6 (17.647)	4 (17.391)	1^b^
*Sleep-Related Bruxism*	11 (7.482)	3 (8.823)	0 (0)	0.265^b^
*Snoring*	17 (11.564)	5 (14.705)	4 (17.391)	1^b^
*Sleep Disturbance, unspecified*	1 (0.680)	1 (2.941)	0 (0)	1^b^

AASM-ICSD, AASM International Classification of Sleep Disorders, n, number; OSA, Obstructive Sleep Apnea; PLM, Periodic Limb Movements; %, percentage. Comparisons between patients with and without a confirmed diagnosis of cMDE were performed using the following tests: ^a^Mann-Whitney U test; ^b^Fisher’s Exact Test; ^c^Chi-squared test; *p<0.05.

We identified 57 patients (38.8%) with a self-administered PHQ-9 total score of ≥10. Among them, 23 patients (40.3%) were diagnosed with a cMDE, with 17 (29.8%) of those meeting the criteria for cMDD and 6 (10.5%) diagnosed with BD, type I. None met the criteria for current BD, type II. Conversely, only 5 patients (5.5%) with a PHQ-9 total score <10 showed a cMDE, resulting in a significantly lower prevalence (Fisher’s Exact Test, p-value <0.0001) (**Table 1**).

The additional comparison of sleep characteristics between patients with PHQ-9 scores ≥ 10 with or without a cMDE, as well as, those with PHQ-9 scores ≥ 10 and cMDD or BD diagnoses, did not reveal any significant differences, except for the percentage of N3 sleep, where patients with a confirmed cMDE spent a significantly shorter proportion of time in this stage (Mann-Whitney U= 240.000, p= 0.049) (**Tables 2**, **3**).

**Table 2:** Comparison of sleep features between patients who scored ≥10 on the PHQ-9 and were diagnosed with a cMDE and those for whom the cMDE was not confirmed.

	Patients with PHQ-9 ≥ 10 without cMDE (n=34)		Patients with PHQ-9 ≥ 10 with cMDE (n=23)		Comparison (Mann-Whitney U test)	
	Median	25^th^-75^th^ percentile	Median	25^th^-75^th^ percentile	U-statistic	p-value
*Total Sleep Time (minutes)*	362.20	(278.25-375.4)	340	(287.0-385.0)	337.500	0.489
*Sleep Efficiency Index*	0.864	(0.742-0.893)	0.837	(0.754-0.926)	229.000	0.388
*Apnea Hypopnea Index events/hour*	9.2	(0.8-30.4)	5.1	(3.2-22.4)	357.500	0.720
*Peripheral Oxygen Saturation <88% events (%)*	0.17	(0-1.75)	0.71	(0.5-3.16)	386.500	0.911
*Respiratory Arousal Index*	4.00	(0.9-16.1)	3.2	(0.8-12.8)	369.000	0.868
*Periodic limb movement arousal index*	0.00	(0.0-4.45)	0	(0.0-1.1)	382.000	0.971
*Spontaneous Arousal Index*	5.20	(2.45-9.1)	6.5	(0.6-8.1)	438.500	0.330
*Total Arousal Index*	12.40	(7.95-24.85)	15.2	(8.1-23.6)	402.500	0.708
*Wake (%)* ^†^	6.543	(4.975-16.004)	9.444	(2.392-15.668)	401.000	0.393
*N1 (%)* ^†^	2.749	(1.061-7.004)	1.998	(18.044-4.202)	348.000	0.951
*N2 (%)* ^†^	46.011	(39.339-57.979)	48.163	(39.134-54.513)	372.000	0.731
*N3 (%)* ^†^	20.290	(8.029-22.690)	14.992	(15.128-27.026)	240.000	0.049*
*REM (%)* ^†^	16.770	(11.511-27.088)	17.67	(11.598-24.796)	384.500	0.573
*REM latency (minutes)*	20.000	(13.625-44.75)	29.5	(10.75-41.125)	382.500	0.597
*Wake After Sleep Onset (minutes)*	24.000	(17.125-55.625)	28.75	(9.0-49.125)	396.500	0.439
*Sleep Onset Latency (minutes)*	130.500	(62-177)	103.5	(85.75-182.75)	210.000	0.305

cMDE, current Major Depressive Episode; n, number; N1, Non-Rapid Eye Movement stage 1; N2, Non-Rapid Eye Movement stage 2; N3, Non-Rapid Eye Movement stage 3; PHQ-9, Patient Health Questionnaire, 9 items; REM, Rapid Eye Movement stage; SD, standard deviation; %, percentage; ^†^The percentage of time spent in each sleep stage was calculated from the moment the subject fell asleep; *p<0.05.

**Table 3:** Comparison of sleep features between patients who scored ≥10 on the PHQ-9 and were diagnosed with cMDD and those with a cMDE diagnosis in BD, Type I.

	PHQ-9 ≥ 10 with cMDE and cMDD (n=17)		PHQ-9 ≥ 10 with cMDE in BD, type I (n=6)		Comparison (Mann-Whitney U test)	
	Median	25^th^-75^th^ percentile	Median	25^th^-75^th^ percentile	U-statistic	p-value
*Total Sleep Time (minutes)*	340.000	(284.5-376.5)	340.950	(256.05-366.675)	59.000	0.609
*Sleep Efficiency Index*	0.831	(0.743-0.871)	0.876	(0.617-0.899)	31.000	0.924
*Apnea Hypopnea Index events/hour*	12.900	(0.8-37.2)	3.450	(1.3-13.775)	63.500	0.400
*Peripheral Oxygen Saturation <88% events (%)*	0.900	(0-8.37)	0.350	(0-0.77)	65.500	0.306
*Respiratory Arousal Index*	3.200	(1.3-18.4)	2.200	(0.4-5.95)	63.500	0.400
*Periodic limb movement arousal index*	0.200	(0.0-5.3)	0.000	(0.0-0.0)	75.000	0.053
*Spontaneous Arousal Index*	6.500	(2.4-9.1)	5.000	(3.05-8.15)	51.500	1.000
*Total Arousal Index*	15.800	(9.8-28.2)	9.100	(6.95-18.525)	69.000	0.227
*Wake (%)* ^†^	9.444	(5.016-15.799)	8.857	(3.174 -26.942)	48.000	1.000
*N1 (%)* ^†^	3.399	(1.457-8.408)	1.115	(0.813-4.492)	69.000	0.134
*N2 (%)* ^†^	49.172	(39.360-58.979)	46.425	(27.792-49.810)	58.000	0.494
*N3 (%)* ^†^	15.970	(10.828-24.380)	13.874	(4.637-17.904)	61.500	0.338
*REM (%)* ^†^	15.619	(9.822-25.787)	24.207	(18.025-29.168)	29.000	0.172
*REM latency (minutes)*	29.500	(20.75-46.875)	23.250	(8.25-37.875)	58.000	0.484
*Wake After Sleep Onset (minutes)*	28.750	(18.375-50.875)	33.000	(12.625-93.875)	46.000	0.914
*Sleep Onset Latency (minutes)*	122.000	(67.0-185.875)	65.000	(61.5-73.5)	48.000	0.257

BD, bipolar disorder; cMDE, current Major Depressive Episode; cMDD, current major depressive disorder; n, number; N1, Non-Rapid Eye Movement stage 1; N2, Non-Rapid Eye Movement stage 2; N3, Non-Rapid Eye Movement stage 3; PHQ-9, Patient Health Questionnaire, 9 items; REM, Rapid Eye Movement; SD, standard deviation; %, percentage; ^†^The percentage of time spent in each sleep stage was calculated from the moment the subject fell asleep; *p<0.05.

The additional comparison of the distribution of sleep disorders and sleep-related symptoms in patients with PHQ-9 scores ≥ 10 and cMDD or BD presenting a cMDE revealed that the most notable finding was a higher prevalence of OSA comorbidity in patients with BD compared to those with cMDD (approximately 67% vs. 53%, respectively; **Table 4**).

**Table 4:** AASM-ICSD-based sleep disorders/sleep-related symptoms distribution in cMDD and BD patients, n (%).

	cMDD (n= 17)	BD, type I (n= 6)
*OSA*	9 (52.941)	4 (66.666)
*Sleep-related hypoxemia disorder*	0 (0)	0 (0)
*PLM disorder*	2 (11.764)	1 (16.666)
*Sleep-related bruxism*	0 (0)	0 (0)
*Snoring*	3 (17.647)	1 (16.666)
*Sleep disturbance, unspecified*	0	0

AASM-ICSD, AASM International Classification of Sleep Disorders; BD, bipolar disorder; cMMD, current major depressive disorder; n, number; OSA, obstructive sleep apnea; PLM, periodic limb movements; %, percentage.

## 4 Discussion

This study is a retrospective sub-analysis of a larger multicenter ongoing study aiming to develop a PSG-based machine-learning algorithm for depression screening in U.S. SCs. The current sub-analysis focused on identifying patients who met the criteria for cMDE among those presenting with clinically significant depressive symptoms, as measured by the self-report PHQ-9 questionnaire, in SCs across the U.S. Our main findings revealed that 38.8% of the patients included in the analysis reported clinically significant depressive symptoms. Among these, 40.3% were diagnosed with a cMDE, with 29.8% meeting the criteria for cMDD and 10.5% diagnosed with BD. In contrast, only about 5% of patients who did not self-report clinically significant depressive symptoms were diagnosed with a cMDE, a significantly lower rate compared to those who reported these symptoms.

Only very few studies involved clinical diagnosis using a structured clinical interview (36, 75, 76) and, to the best of our knowledge, only another study aimed to determine the prevalence of clinical depression after depressive symptoms screening in a sample of patients with OSA (36). This study reported that 28% of OSA patients presented mild to severe depressive symptoms and 80.2% of those met the criteria for a depressive disorder according to ICD-10 (36). Compared to our study, this research reported a lower rate of clinically significant symptoms but twice the rate of clinical depression among those screened for mild to severe depressive symptoms. To assess the replicability of Acker’s findings, we focused our analysis on patients with OSA who were screened using a PHQ-9 cut-off score of ≥ 10. In this subsample, the rate of clinically significant symptoms was higher than in Acker’s study. Specifically, 34 out of 103 patients (33%) had PHQ-9 scores above the cut-off. However, among these 34 patients, 24 (70%) were identified with cMDE, and 20 out of those 24 (83.3%) were confirmed to have cMDD. These results demonstrate similar rates of cMDD diagnosis in our group compared to the previous study (36). The differences in depressive symptom rates may be due to differences in methodology, such as a different population (OSA patients from a European single-sleep center (36) *vs* sleep disorders or symptoms from multiple U.S. sleep centers), the use of different screening questionnaires with more restrictive and specific screening criteria (BDI II ≥ 14 and WHO-5 ≤ 13 (36) *vs* PHQ-9 ≥10).

Similarly to other studies investigating the prevalence of depression in sleep disorder samples (36, 45, 48, 49) our study reported about 5-fold rates of cMDE compared to the general population (5–7) underlying the correlation between sleep disorders and depression. Our finding, along with the aforementioned studies, highlights the need for depressive symptoms screening in SCs and diagnosis confirmation assessment in psychiatric settings.

Other studies in U.S. primary care settings showed that depression screening increased the odds of depression diagnosis. Although only 4-5% of visits included depression screening, those visits were up to nine times more likely to result in a depression diagnosis compared to visits without screening (15, 77). Moreover, similarly to our results, when depression screening occurred in primary care settings, 47% resulted in a new depression diagnosis (13).

Depression screening may also help in identifying those individuals who are at high risk of suicide. For example, item 9 of the PHQ-9 assesses the presence of suicidal ideation and has been shown to be a reliable predictor of suicide risk over time (78). Since several DSM-5-based sleep disorders (e.g., insomnia, nightmares) have been linked to suicide risk, the implementation of depression/suicide screening and patient safety protocols becomes crucial. In line with this, recent recommendations suggest using a depression screening tool (e.g., PHQ-9), followed by an evidence-based assessment with high sensitivity and specificity to detect both imminent and medium-term suicide risk (79, 80).

The comparative analysis between patients with clinically significant depressive symptoms, with or without a cMDE, revealed no significant differences in most sleep features, except for the percentage of N3 sleep. Patients with a confirmed cMDE spent a significantly shorter proportion of time in this stage. Overall, our findings do not fully align with existing literature. Previous studies have reported that individuals with depression exhibit objective alterations in sleep physiology compared to non-depressed individuals, including sleep fragmentation, prolonged sleep latency, increased frequency and duration of the awakenings, increased REM latency and density (81–85). However, in our sample, the absence of significant differences in these parameters may suggest that the overall burden of depressive symptoms negatively impacts sleep characteristics, regardless of whether the criteria for cMDE are met.

Our findings did, however, reveal a significantly shorter proportion of N3 sleep in patients with a confirmed cMDE. Previous studies have associated depressive disorders with reduced sleep depth, particularly alterations in non-rapid eye movement (NREM) sleep, including a decrease in slow-wave sleep (SWS) during the first NREM period (84, 86–88). However, these findings remain inconsistent due to small sample sizes and methodological variations, with some studies reporting no differences in SWS or even an increase in patients with depression compared to controls (89).

Moreover, given the presence of comorbid sleep disorders and sleep-related symptoms in patients with confirmed cMDE, findings on depression alone may not be directly generalizable to our sample.

The comparative analysis between patients with cMDD and BD, type I did not find significant differences in sleep characteristics. To the best of our knowledge, few studies have directly compared sleep features between mood disorders and BD. A recent retrospective exploratory cross-sectional study of PSG recordings (90) examined sleep differences between patients with recurrent depressive disorder (RRD) and BD during full or partial remission. Consistent with our findings, this study reported no significant differences in sleep macrostructure, with both BD and RRD patients exhibiting similar sleep duration and arousal levels. The only notable difference—despite varying prevalence rates—was a higher AHI in BD patients compared to those with RRD, reflecting the greater prevalence of OSA in BD (50.8% vs. 29.3% in their study; approximately 67% vs. 53% in ours). As some authors suggested, the lack of macrostructure differences in affective disorders may be due to the lack of specific neurophysiological markers underlying common neurobiological mechanisms (91). Furthermore, medical and sleep-related comorbidities in our sample may have masked the specific effects of depression on sleep physiology.

Our study presents some limitations. The data analyzed in this study was derived from a sub-analysis of a larger ongoing study. Although the sample size is adequate, it may not be fully generalizable to the broader sleep disorder population. Future studies could include more than thirteen SCs, covering a wider geographic area, to obtain a more representative sample.

Country-specific policies may have introduced a bias in referring patients to SCs, potentially influencing psychiatric prevalence estimates by including only those with severe OSA. Furthermore, patients with severe mental and physical conditions might be more appropriately assessed in a home environment, introducing additional bias into the representativeness of the sample. Additionally, cultural and socioeconomic factors may have contributed to the lower prevalence of certain racial groups, such as Hispanics (approximately 11%), which, in contrast, show higher representation in the geographical areas covered by our data collection. On the other hand, other minority groups, such as Asian, Native American, and Native Hawaiian, may be underrepresented in our sample due to their lower overall representation in the U.S. general population (92). However, despite the relatively low prevalence of these minority groups, our sample revealed a higher proportion of Asians with a confirmed clinically diagnosed cMDE compared to those without such a diagnosis (**Table 1**). Some studies have reported that while the prevalence of depression among Asian Americans is lower than in other ethnic/racial groups, they are less likely to receive a diagnosis due to cultural barriers (93) and are less likely to seek treatment, which is often of suboptimal quality (94). This may suggest that the higher prevalence of cMDE among Asians in our sample could reflect a bias, potentially driven by greater clinical severity that requires more intensive clinical attention. Such individuals may be more likely to seek or receive care, contributing to the higher observed prevalence of cMDE, particularly when comorbid with sleep disorders.

Our sample may also be influenced by a selection bias related to socioeconomic status. The absence of income data prevents us from assessing the representativeness of our sample, particularly regarding patients with lower socioeconomic status, where depression and sleep disorders are typically overrepresented. Research consistently shows that lower socioeconomic status is associated with higher rates of depression and poor sleep quality due to factors such as financial stress, limited healthcare access, and higher exposure to psychosocial stressors (95). Additionally, individuals with higher socioeconomic status are more likely to seek medical care, receive a diagnosis, and have greater access to healthcare services. This can lead to a potential overrepresentation of those with better healthcare access in clinical samples (96). Accounting for income would help identify potential biases arising from greater access to medical care among individuals who can afford healthcare services.

Additionally, we excluded subjects older than 75 years. The literature lacks a clear consensus on the age at which significant changes in HRV occur. Some studies suggest that such changes may begin around age 60 (97), particularly when associated with depression (98). In alignment with the selection criteria used in the larger ongoing algorithm development study, we adopted a less restrictive cutoff to avoid limiting the algorithm’s applicability to a narrower population typically seen in SCs, where the rate of cMDE may be increased. Indeed, our data show that 5 out of 22 individuals (approximately 23%) aged over 60 reported a cMDE, a notably higher rate compared to younger individuals (<60 years old), in whom the cMDE rate was about 15%.

Despite thorough training in MINI administration, the lack of role-playing with the same clinical cases did not allow for estimating the statistical agreement among the involved nurses. Consequently, the diagnostic reliability in our study may be influenced by potential uncontrolled biases, particularly due to the lack of verification of the nurses’ diagnostic performance. This issue is especially relevant in our population, where the risk of misdiagnosis is elevated due to the overlap between symptoms of cMDE and sleep disturbances. Moreover, the nurses did not take into account the presence of psychiatric history, including past MDE/MDD and the presence of psychotropic therapy. To avoid bias and improve interrater reliability in future studies, as suggested by the literature (99–101) the assessment should be conducted through consensus between two or more clinicians with expertise in affective disorders, incorporating both medical and psychiatric history. Additionally, it should involve more structured training to enable statistical evaluation of diagnostic agreement.

As a final consideration, our sub-analysis had a small sample size, which may have reduced statistical power and increased the risk of a Type II error, potentially leading to non-detection of true differences in most of the additional comparative analyses. These results may also be influenced by another limitation—the lack of information on pharmacotherapy, particularly psychotropic treatments. The potential use of benzodiazepines and antidepressants could have altered sleep patterns, potentially masking differences between patients with cMDD and BD.

Despite these limitations, there is consensus that due to the high comorbidity between sleep disorders and clinical depression, screening for depression in sleep services should be the initial step in the diagnostic process (36, 37, 102, 103). Detecting comorbid depression can enhance continuity of care between SCs and primary care. When depression is identified, it is crucial for sleep clinicians to inform the patient about the screening results and integrate them into a coordinated clinical pathway. This should involve communication with the patient’s general practitioner or referring clinician to initiate further evaluation, including a diagnostic consultation with a mental health professional for a comprehensive assessment to confirm or rule out an MDE. Even in cases of false-positive detection, identifying a depressive burden may indicate the presence of subclinical conditions that could benefit from support and treatment. Conversely, false-negative results prevent patients from receiving appropriate care, leading to persistent distress, functional impairment, increased morbidity and mortality, and ultimately, a reduction in productive years of life (104), highlighting the crucial role of depressive screening.

This approach fosters a comprehensive understanding of the patient’s clinical condition, enables tailored treatments, reduces the risk of errors, and improves therapeutic outcomes.

### 4.1 Future perspectives

Some studies have reported that between 2010 and 2020, the incremental economic burden of adults with MDD increased by 37.9%, rising from $236.6 billion to $326.2 billion (105). Other studies have reported that routine screening schedules are cost-effective compared to the baseline, with a gain ranging from $11,134 to $34,065 per quality-adjusted life year (106). Thus, integrating depression screening into clinical practice has been encouraged. The ultimate goal is to make the screening process as routine and robust as possible, ensuring widespread effectiveness and reliability. Specifically, an automated depression screening tool based on automatized psychometric questionnaires like the PHQ-9 offers automatic and time-saving scoring (107).

Moreover, the advancement of tools based on evidence-based physiological biomarkers of depression could enhance screening reliability by reducing the assessment burden, mitigating physicians’ subjective biases, minimizing the risk of misdiagnosis, and improving detection rates of depression. Due to the powerful link between sleep and mood regulation, PSG research has largely contributed to investigating psychobiological mechanisms of depression, especially those related to imbalanced sleep architecture and autonomic nervous system function. The literature extensively documents changes in objective sleep architecture associated with depression, including sleep fragmentation, disinhibition of rapid eye movement (REM) sleep, and alterations in non-REM sleep, notably decreased stage N2 and slow-wave sleep, which are considered hallmarks of depression (82). Consequently, these parameters could serve as depression biomarkers, making the PSG suitable for MDD screening in patients referring to SCs for sleep disturbances. Thus, automated tools may represent a solution to the challenge of conducting time-consuming traditional assessments in non-psychiatric clinical settings.

## Data Availability

The datasets presented in this article are not readily available because the dataset is Medibio Limited property. Requests to access the datasets should be directed to massimiliano.grassi@medibio.com.
